# Genotype–Phenotype Correlations of Nance–Horan Syndrome in Male and Female Carriers of a Novel Variant

**DOI:** 10.3390/genes16010091

**Published:** 2025-01-16

**Authors:** Olivia A. Zin, Luiza M. Neves, Fabiana L. Motta, Daltro C. Junior, Daniela P. Cunha, Bruna N. S. Agonigi, Jocieli Malacarne, Ana Paula S. Rodrigues, Gabriela D. Rodrigues, Maria Luisa C. Tinoco, Dafne D. G. Horovitz, Adriana B. Carvalho, Andrea A. Zin, Zilton F. M. Vasconcelos, Juliana M. Ferraz Sallum

**Affiliations:** 1 Ophthalmology Department, Federal University of São Paulo, São Paulo 04039-032, Brazil; olivia.zin@unifesp.br (O.A.Z.);; 2 Instituto Brasileiro de Oftalmologia, Rio de Janeiro 22250-040, Brazil; 3 Instituto Nacional de Saúde da Mulher, da Criança e do Adolescente Fernandes Figueira-Fundação Oswaldo Cruz, Rio de Janeiro 22250-020, Brazilzilton.vasconcelos@fiocruz.br (Z.F.M.V.); 4 Ophthalmology Department, Universidade do Estado do Rio de Janeiro, Rio de Janeiro 20551-030, Brazil; 5 Instituto de Genética Ocular, São Paulo 04552-050, Brazil; 6 Instituto Nacional de Cardiologia, Rio de Janeiro 22240-006, Brazil; 7 Instituto Catarata Infantil, Rio de Janeiro 22250-040, Brazil

**Keywords:** Nance–Horan syndrome, congenital cataract, pediatric cataract, X-linked disease

## Abstract

Background: Nance–Horan syndrome (NHS) is a rare, frequently underdiagnosed, X-linked disease caused by mutations in the NHS gene. In males, it causes bilateral dense pediatric cataracts, dental anomalies, and facial dysmorphisms. Females traditionally have a more subtle phenotype with discrete lens opacities as an isolated feature. The objective of this case report is to describe a novel variant in NHS, as well as to discuss genotype–phenotype correlations. Methods: Whole-exome sequencing was performed in 3 affected individuals (2 males and 1 female) with pediatric cataracts from the same family, as well as in 2 unaffected members from the same family. Ophthalmological and clinical genetic evaluations were conducted. Results: The likely pathogenic variant c.3333del (p.Phe1111Leufs*9) was found in all affected individuals, as well as in one unaffected female family member. Our family was initially diagnosed with isolated hereditary cataracts, but only after the sequencing results was the phenotype revealed, with the systemic features being identified. Conclusions: This reinforces the importance of genetic testing of bilateral familial pediatric cataracts, especially since systemic features such as dental anomalies and intellectual disability may take years before they develop. Not only did genetic testing help to identify extraocular features, but it also made possible accurate family counseling essential in all pediatric cataract cases.

## 1. Introduction

Nance–Horan syndrome (NHS) (OMIM: 302350) is a rare X-linked disease characterized by ocular features, dental anomalies, and facial dysmorphisms. The main ophthalmological finding is dense congenital nuclear cataracts, but microphthalmia, nystagmus, and strabismus are also observed. Dental anomalies include screwdriver-shaped incisors, supernumerary teeth, diastema, and tapered premolar/molar cusps. Individuals may have a long, narrow face, a bulbous nose, and anteverted pinnae, whilst intellectual disability can also be present [1,2].

Even though the syndrome has many distinct characteristics, its diagnosis can present a challenge since cataracts, the earliest finding, may have many causes in infants, such as isolated hereditary cataracts or even be secondary to infectious diseases. Other features of NHS, such as dental anomalies and intellectual disabilities, which could guide the differential diagnosis, may take years before they go noticed.

Hemizygous males traditionally manifest the full spectrum of features with dense congenital nuclear cataracts, while heterozygous females generally present with posterior Y sutural cataracts and other mild features [3]. Very rarely, asymptomatic female cases have been described [4].

Nance–Horan is a rare syndrome, and the only other South American patients reported are of Mexican ancestry [5,6]. Most pathogenic variants are nonsense, usually caused by frameshift mutations, leading to premature stop codons and truncated proteins [7].

The purpose of this report is to describe a novel nonsense frameshift variant causing Nance–Horan syndrome in a Brazilian family. Genotype–phenotype correlations are examined, taking into account X-linked inheritance and the expected manifestations based on gender. In addition to the male patients, this report also includes rare manifestations in female carriers.

## 2. Materials and Methods

### 2.1. Study Design

**Figure 1:**
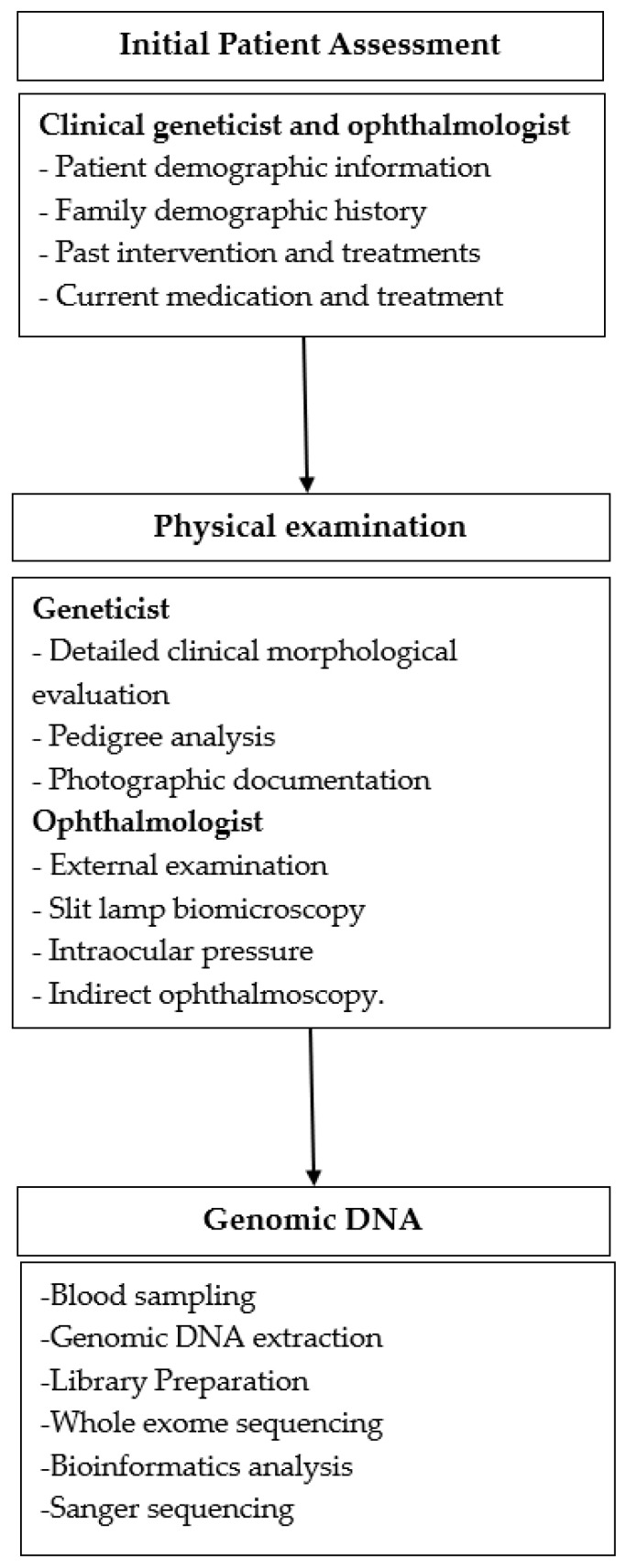
Flowchart of study design.

This case report provides a clinical description and next-generation sequencing analysis of five individuals from a family with a history of pediatric cataracts. Among the five individuals, three were symptomatic, and two were asymptomatic. The study design is summarized in Figure 1. This case report is part of a larger research project whose objective is to identify genes and variants associated with pediatric cataracts, analyzing the impact on prognosis, clinical evaluation, and family counseling. The project is funded by the Brazilian National Program of Support to the Health Assistance of the Person with Deficiency (PRONAS/PCD). The methodology described here has already been reported elsewhere [8,9].

### 2.2. Clinical Evaluation

An ophthalmic examination was conducted by an ophthalmologist using slit-lamp biomicroscopy, intraocular pressure (IOP) measurement, and indirect ophthalmoscopy for all individuals. Photographic documentation of external ocular features and facial characteristics was also completed. A clinical geneticist performed a genetic evaluation, which included a review of family history, pedigree analysis, and any relevant systemic findings. The history of congenital TORCH infections (toxoplasmosis, rubella, cytomegalovirus, herpes simplex, syphilis, varicella zoster, and Zika), as well as the use of corticosteroids and ocular trauma, were ruled out.

### 2.3. Genomic DNA

Peripheral blood samples were collected in EDTA tubes. PureLink^®^ Genomic DNA Mini Kit Thermofisher (Waltham, MA, USA) was used to extract genomic DNA from peripheral blood leukocytes according to the manufacturer’s protocol [7]. Invitrogen Qubit^®^ 4 Fluorometer Thermofisher (Waltham, MA, USA) was used to determine DNA concentration in samples. To assess DNA purity, a spectrophotometer NanoDrop^®^ 2000 Thermofisher (Waltham, MA, USA) was used to evaluate the ratio of the absorbance at 260/280 nm (average of 1.90 for all samples) and 260/230 nm (average of 1.91 for all samples). DNA samples were stored at 4 °C before use.

### 2.4. Library Preparation and Whole-Exome Sequencing

Illumina DNA Prep with Enrichment (16 samples) was used to prepare DNA libraries according to the manufacturer’s instructions (Illumina, Inc., San Diego, CA, USA). The Illumina Exome Panel (San Diego, CA, USA) was used, which covers 37.5 Mb of coding content (≥99% of RefSeq, CCDS, ClinVar, and ACMG pathogenic/likely pathogenic variants, COSMIC Cancer Gene Census). Sequencing was performed using the NVSEQ 6000 S4 Rgt Kit v1.5 Illumina Inc. (200 cyc) (San Diego, CA, USA). During library preparation, DNA fragments of 400 bp long on average were evaluated using the Bioanalyzer by Agilent (Santa Clara, CA, USA).

### 2.5. Bioinformatics Analysis

Sequencing data were processed using the nf-core pipeline Sarek aligned to the human reference genome GRCh38 [10]. The generated VCF files were analyzed using Franklin^®^ by the Genoox platform (Israel), and the genetic variant calls were performed against the reference sequence of hg38 from the University of California Santa Cruz (UCSC) (Santa Cruz, CA, USA) Genome Browser. The analysis strategy started with a virtual panel based on Human Phenotype Ontology (HPO), where 658 genes were found related to cataracts in general. Genogram trio analysis was performed on affected and unaffected family members using the trio analysis tools available on Franklin^®^. During variant interpretation, we considered allele frequency using the Exome Aggregation Consortium database (ExAC), the 1000 Genomes Project database, gnomAD, and ABraOM, an online archive of Brazilian mutations [11]. Thirteen predictors were considered for pathogenicity: REVEL, BayesDel_addAF, DANN, DEOGEN2, EIGEN, FATHMM-MKL, LIST-S2, M-CAP, MVP, MutationAssessor, MutationTaster, SIFT, and PrimateAI. The clinical significance of variants was evaluated with ClinVar, the Polymorphism database (dbSNP), and the Human Gene Mutation Database (HGMD). Cat-Map [12], an online chromosome map and reference database for cataracts in humans and mice, was also searched for previous variant descriptions and clinical associations. Variant naming was based on the reference sequence NM_001291867.2.

### 2.6. Sanger Sequencing

In order to confirm the variant identified by clinical exome analysis, PCR amplification and bi-directional direct Sanger sequencing were performed using the oligonucleotide primers [Forward: 5′CCACCACCCCATCTCTTCCT3′ and Reverse: 5′CCGTTGTGAGTGTGCTTTCC3′]. Primers and PCR products were purified using PureLink^®^ (Invitrogen™, Carslbad, CA, USA)) and sequenced on an automated sequencer (ABI 3730 Genetic Analyzer, Applied Biosystems)(Foster Ciry, CA, USA). Results were interpreted by BioEdit software (Manchester, UK).

## 3. Results

### 3.1. Ophthalmological Exam

**Figure 2:**
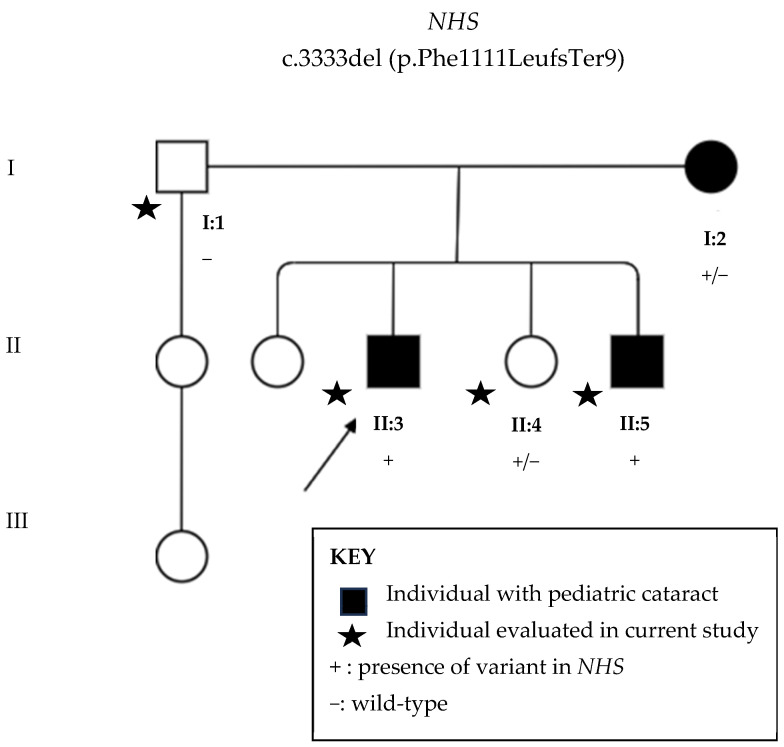
Family pedigree with three generations depicted. Circles indicate women and squares men. Black circles and squares indicate individuals with pediatric cataracts. Stars indicate family members evaluated in the current study. +: presence of variant c.3333del (p.Phe1111Leufs*9) in *NHS* gene located in X chromosome, −: wild type.

**Figure 3:**
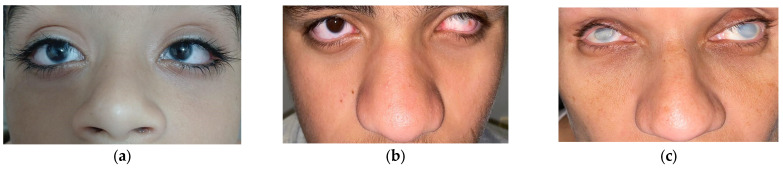
External features of Nance–Horan syndrome patients: (**a**) Bilateral clear corneas, microcornea, and strabismus in male II:3 (**b**) Bulbous nose, bilateral microcornea with clear right cornea and right opacity in male II:5 (**c**) Bulbous nose, bilateral microcornea with opaque corneas in female individual I:2.

The proband (Figure 2, individual II:3) was examined at the age of 15 years and was found to be pseudophakic, exhibiting strabismus and nystagmus. Visual acuity was 20/60 in both eyes. A diagnosis of congenital cataracts was made at 2 months, and a facectomy was performed at age 1. Slit-lamp biomicroscopy revealed clear corneas bilaterally (Figure 3a), normal intraocular pressure (IOP), and fundoscopy showed no abnormalities.

His elder brother (Figure 2, individual II:5), aged 18 years, was diagnosed with cataracts at 2 months and underwent surgery at 6 months old. Visual acuity was 20/125 in both eyes. Slit-lamp examination (Figure 3b) revealed a clear right cornea with a punctiform pupil, while the left cornea was completely opaque, with calcium deposits, making the anterior chamber unidentifiable. Fundoscopy was not visible in either eye. IOP was normal.

The mother (Figure 2, individual I:2), aged 45 years, was diagnosed with congenital cataracts at 1 month old and underwent surgery at 4. Currently, she has total bilateral corneal opacities (Figure 3c), with both the anterior chamber and fundoscopy being unobservable. IOP was normal.

There is limited information regarding the phenotypes of cataracts in these individuals, as they have already undergone surgery and previous medical records are unavailable. The father (I:1) and daughter (II:4) showed no ophthalmic abnormalities. All five evaluated family members denied any systemic conditions.

### 3.2. Next-Generation Sequencing

Whole-exome sequencing revealed in all examined members, except in the unaffected father, the novel likely pathogenic variant c.3333del (p.Phe1111Leufs*9). That is, the sons (II:3 and II:5) presented the variant in hemizygosis, whilst the affected mother (I:2) and unaffected daughter (II:4) were heterozygotic for the variant.

### 3.3. Sanger Sequencing

**Figure 4:**
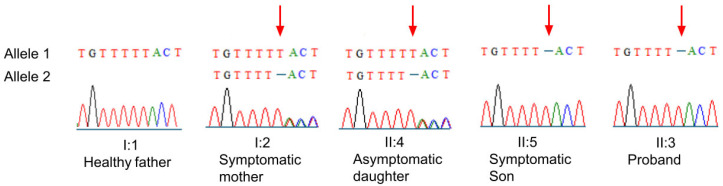
Sanger sequencing results confirm the presence of novel variant c.3333del (p.Phe1111Leufs*9) in the *NHS* gene located on the X chromosome in all family members identified previously by next-generation sequencing. Red arrows point to the location of variant in affected individuals.

Sanger sequencing (Figure 4) confirmed the presence of the variant in all affected individuals, as well as in the unaffected daughter (individual II:4).

### 3.4. Systemic Findings

**Figure 5:**
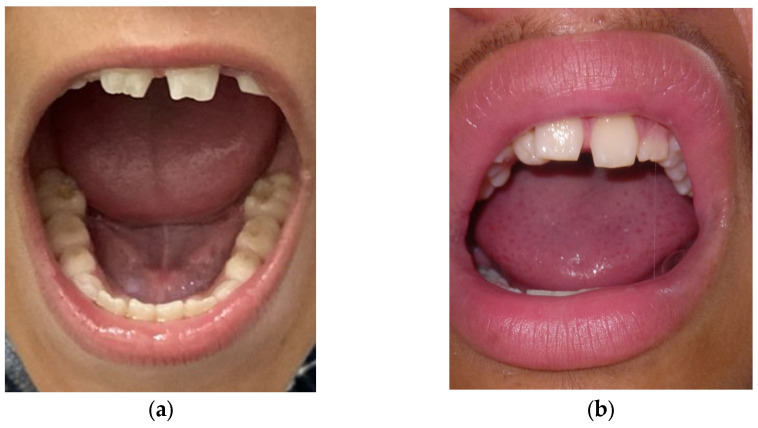
Teeth alterations in Nance–Horan syndrome: (**a**) Screwdriver-shaped incisors and diastema in individual II:3; (**b**) screwdriver-shaped lateral incisors and diastema in individual II:5.

The individuals were initially classified as having isolated familial cataracts but were reevaluated following the next-generation sequencing results. A search for dental anomalies was then made, and these were discovered in both affected males, II:3 (Figure 5a) and II:5 (Figure 5b), but not the unaffected daughter (II:4). Affected mother (I:2) had prosthetic teeth due to an accident that caused loss of her teeth, so her dental evaluation was impaired. Both males exhibited diastema (a gap between the teeth), mulberry molars, and screwdriver-shaped incisors, with the proband having both central and lateral incisors affected, while only the lateral incisors were affected in his brother.

Facial features were also reevaluated upon receiving the sequencing results, and individuals I:2 and II:5 had a bulbous nose, as well as a long and narrow face, characteristic of NHS. None of the features were observed in the unaffected female (II.4).

The clinical features of patients are summarized in Table 1.

## 4. Discussion

The *NHS* gene is expressed in a variety of tissues, including the lens, brain, craniofacial mesenchyme, and dental primordia, as reported by Brooks et al. [13] and Burdon et al. [14]. The lens’ shape and transparency depend on the morphology and migration of lens fiber cells, along with membrane remodeling and intercellular interactions. The NHS protein acts as an actin regulatory protein, overseeing the activity of actin proteins to build cytoskeletal structures and facilitate cell adhesive interactions that are crucial for lens fiber elongation and differentiation, as described by Brooks [15] and Rao [16]. Disruption in these mechanisms can result in loss of lens transparency and the development of cataracts.

The novel variant herein described c.3333del (p.Phe1111Leufs*9) is located in a region of *NHS*, where the majority of pathogenic mutations are found. According to the American College of Medical Genetics and Genomics (ACMG) [17], it is classified as likely pathogenic because it is a null variant in a gene where the loss of function is a recognized mechanism for disease (PVS1) and is absent from population databases (PM2). Most pathogenic variants identified in *NHS* are nonsense, primarily resulting from frameshift mutations that lead to premature stop codons and truncated proteins [12]. Therefore, the fact that this variant is a frameshift mutation located in a region of the gene where many loss-of-function variants have been described reinforces its pathogenicity. Also, it is also not found in population databases, such as gnomAD, which further supports the genotype–phenotype association presented here.

In women, traditional NHS phenotypes typically include posterior Y sutural cataracts [3,18,19], though there is significant clinical variability. On one end of the spectrum, severe congenital cataracts have been reported in females [6,20,21], as the elderly female here reported, while others exhibit milder ocular and systemic symptoms [2,19,22]. Truly asymptomatic female carriers, lacking characteristic NHS features as described here, have been documented by Huang et al. [4], who concluded through molecular studies that symptom variability in female carriers is due to nonrandom X chromosome inactivation.

X-linked inheritance often results in more pronounced clinical manifestations in hemizygous males compared to heterozygous females. Heterozygous females can be asymptomatic carriers or manifest mild symptoms. Such variability may be the result of X-chromosome inactivation in females, where one of the two chromosomes is randomly inactivated in somatic cells. If the X chromosome without the pathogenic variant is disproportionately inactivated, the chromosome with the pathogenic variant will be greatly expressed. Therefore, skewed X-inactivation can cause the variability of clinical manifestations in females [23,24].

Gomez-Laguna et al. [6] also described a family where both mother and daughter exhibited severe ocular and systemic symptoms. Cytogenetic and molecular studies confirmed a balanced rearrangement, preferential inactivation of the normal X chromosome, and undetectable NHS RNA expression. Thus, it is hypothesized that the symptom variability between our severely affected mother and her asymptomatic daughter, despite sharing the same genotype, could be due to skewed X inactivation. This family was initially diagnosed with isolated hereditary cataracts, but subsequent next-generation sequencing (NGS) results revealed associated systemic features. This underscores the importance of genetic testing in familial pediatric cataract cases. Systemic features, such as intellectual disability and dental abnormalities in NHS, may take years to manifest, presenting an even greater diagnostic challenge. Additionally, Lenassi et al. [25] demonstrated the clinical utility of diagnostic testing in infants with various inherited eye disorders. Genetic testing in pediatric cataract patients prevented unnecessary further diagnostic procedures in 50% of probands and initiated monitoring for extraocular features in 12%. This represents one of the highest rates of clinical utility compared to other pediatric inherited ocular diseases, including traditionally tested retinal dystrophies, further emphasizing the value of genetic testing in pediatric cataracts. Indeed, this family had been investigated for congenital syphilis for their teeth appearance, the main dental differential diagnosis.

Genetic testing not only facilitated the identification of extraocular features but also enabled accurate family genetic counseling, which can be particularly challenging in diseases with X-linked inheritance. In such cases, mothers may exhibit mild symptoms while their male offspring experience more severe manifestations. For instance, Lowe syndrome is another X-linked condition characterized by dense congenital nuclear cataracts in boys, while affected mothers may only show mild lens opacities as isolated features. This syndrome is severe, with affected males often experiencing hypotonia, renal failure, and a life expectancy of approximately 40 years [26]. By conducting genetic testing in females with the seemingly benign phenotype of early cataracts, it becomes possible to provide appropriate familial counseling regarding this serious syndrome in males.

In all cases of pediatric cataracts, early diagnosis, and treatment are crucial to minimize the risk of amblyopia and significantly enhance visual outcomes. Most unilateral cases are isolated, nonhereditary, and lack systemic associations, so extensive laboratory investigations are generally not necessary [27]. However, this is not true for bilateral pediatric cataracts, where traditional investigations include tests for serum calcium and phosphorus, blood sugar levels, urine screening for reducing substances, erythrocyte assays, and TORCH titers [28]. Recently, measuring ferritin levels in pediatric cataract patients has been recommended due to the rising number of identified hereditary hyperferritinemia cataract syndrome cases [8]. Given that most bilateral cataracts in children have a genetic basis, genetic molecular investigation should also be conducted [28]. This investigation can be cost-prohibitive under public health systems scenarios. However, a recent Brazilian study estimated the cost of genetic testing per exam for pediatric cataracts under the Brazilian Governmental Health System, showing that with appropriate clinical indications, genetic testing for pediatric cataracts could be affordable and have a high clinical utility [29]. An accurate diagnosis allows for the prevention of these diseases in future generations through genetic counseling, prenatal diagnosis, and even pre-implantation genetic diagnosis [30].

## 5. Conclusions

In conclusion, this case report highlights the significance of genetic testing in diagnosing Nance–Horan syndrome (NHS). The identification of the novel variant c.3333del (p.Phe1111Leufs*9) in affected individuals underscores the need for comprehensive genetic evaluations in families with bilateral pediatric cataracts. Our findings demonstrate that genetic testing not only aids in uncovering systemic features that may not be immediately apparent but also facilitates accurate family genetic counseling.

## Figures and Tables

**Table 1:** Clinical features of male and female patients with one *NHS* likely pathogenic variant.

		Male		Female	
Disease Phenotype		II:3	II:5	I:2	II:4
Ocular features	Pediatric cataract	+	+	+	−
	Microcornea	+	+	+	−
	Corneal opacity	−	+	+	−
	Strabismus	+	+	+	−
	Nystagmus	+	−	−	−
Facial features	Long−narrow face	−	−	−	−
	Bulbous nose	−	+	+	−
	Anteverted pinnae ^1^	+	+	−	−
Dental features	Screwdriver-shaped incisors	+	+	N/A	−
	Mulberry molars	+	+	N/A	−
	Diastema ^2^	+	+	N/A	−
Other	Intellectual disability	+	+	−	−

^1^ Ear flaps, or the outer part of the ear. ^2^ Gap between teeth. +: Presence of clinical feature. −: absence of clinical feature. N/A: Not applicable, since mother had prosthetic teeth.

## Data Availability

Data are available on the public online database Cat-Map and from related publications and supplements. These data can be found here: https://cat-map.wustl.edu/, accessed on 22 May 2023.
